# INNOVATIVE GERIATRICS EDUCATION FOR STUDENTS AND PROFESSIONALS

**DOI:** 10.1093/geroni/igae098.2164

**Published:** 2024-12-31

**Authors:** Erin Emery-Tiburcio

**Affiliations:** Chair

## Abstract

Training in working with older adults is lacking across health care disciplines, and in interprofessional education, both at the student and licensed professional levels. This symposium will describe five unique and highly diverse programs to educate students and professionals about elements of providing optimal health care to older adults. The Extension for Community Healthcare Outcomes (ECHO) Geriatrics program at the University of Washington engaged interprofessional health professions students to participate in monthly learning sessions about age-friendly care and post-session debriefs. The University of Louisville engaged social work students in their highly successful FlourishCare curriculum (FCC), which includes online modules, workshops, interprofessional case management experiences, case conceptualization meetings, Project ECHO, and more. The 4Ms STOP and WATCH Program at St. Louis University provides education and experiential learning for Certified Nursing Assistants (CNAs) in nursing homes. Virginia Commonwealth University trained Emergency Medical Services (EMS) providers on foundational knowledge and skills in care of older adults living with dementia, including advance care planning needs. Finally, the 4Ms framework of an Age-Friendly Health System (What Matters, Medication, Mentation, Mobility) was adapted by Rush University for use by behavioral health providers, who provided training to interprofessional Community Mental Health Center clinicians. Each of these programs has demonstrated significant positive impact and serve as models for training in geriatrics.

